# The role of nutrition‐sensitive agriculture combined with behavioral interventions in childhood growth in Ethiopia: An adequacy evaluation study

**DOI:** 10.1002/hsr2.524

**Published:** 2022-03-03

**Authors:** Tefera Chane Mekonnen, Sisay Eshete Tadesse, Yeshimebet Ali Dawed, Nigus Cherie, Hunegnaw Abebe, Getachew Shumye, Foziya Mohammed, Ahmed Hussien

**Affiliations:** ^1^ Nutrition and Dietetics Department School of Public Health, College of Medicine and Health Sciences, Wollo University Dessie Ethiopia; ^2^ Reproductive and Family Health Department School of Public Health, College of Medicine and Health Sciences, Wollo University Dessie Ethiopia; ^3^ Departments of Animal Science College of Agriculture, Wollo University Dessie Ethiopia; ^4^ Department of Plant Science College of Agriculture, Wollo University Dessie Ethiopia

**Keywords:** 1000 days, Ethiopia, behavioral change, child‐linear growth, egg consumption, nutrition education, nutrition‐sensitive agriculture intervention

## Abstract

**Objective:**

The study aimed to investigate the role of nutrition‐sensitive and specific interventions along with nutrition education on child stunting during the first 1000 days in Ethiopia.

**Methods:**

An adequacy evaluation study was used to see changes between the baseline and end‐line data after following for 1 year. A sample of 170 mother‐child pairs who had a 1‐year followed up was used to detect differences. We performed structural equation modeling to elucidate changes in feeding behaviors, socioeconomic status, water, sanitation and hygiene on child linear growth. Furthermore, the independent effect of covariates on child linear growth was handled using a general linear model.

**Results:**

A total of 170 and 270 mother‐child dyads were interviewed at baseline and end‐line surveys, respectively. After about 1 year of intervention, the annual rate of stunting prevalence declined from 29.3% (95% confidence interval [CI] = 18.6, 42.7) to 16.4% (95% CI = 10.7, 24.2). There was a significant change in the mean of length‐for‐age *Z*‐score which changed from −1.18 to −0.45 (*P* < .034). Adjusting for the different constructs of the health belief model, child sex, age, feeding behaviors, and dietary diversity, one egg consumption per day was responsible for the most significant variability explained (36%) for stunting reduction.

**Conclusions:**

Sustainable access to egg consumption for children below 2 years experienced a substantial reduction in childhood stunting. A combination of nutrition‐sensitive agricultural and direct nutrition interventions along with behavioral‐based education is a sustainable strategy in reducing and preventing child growth from faltering in the early life stages.

## Graphical abstract

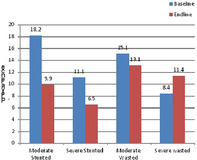

**Abbreviations**
ADA: agricultural development army
ASF: animal source food
CDDS: Child Dietary Diversity Score
CFA: confirmatory factor analysis
CFI: comparative fit index
FAO: food and agriculture organization
GFI: goodness of fit index
HBM: health belief model
HEW: health extension worker
IYCF: infant and young child feeding
LAZ: length‐for‐age *Z*‐score
MAD: minimum acceptable diet
MDD‐W: minimum dietary diversity for women
MMF: minimum meal frequency
MUAC: mid upper arm circumference
RMSEA: root mean square error of approximation
SEM: structural equation modeling
SRMR: standardized root mean squared residual
TLI: Tucker‐Lewis index
UNICEF: United Nations International Children's Fund
WASH: water, sanitation and hygiene
WDA: women development army

## 1 INTRODUCTION

Malnutrition among children under 5 years of age is still a widespread problem in many developing countries.^1^ Child stunting, an indicator of chronic malnutrition, is a global public health problem and is both a symptom of past deprivation and a predictor of future poverty.^2^ Failure to access essential nutrients during pregnancy and the first 2 years of life undermines the survival, growth, and development of children.^3^, ^4^

The number of stunted children has declined in all continents, except in Africa.^2^ The etiology of early childhood malnutrition is complex, involving interactions between parental feeding practices, dietary intakes, and nutrient absorptive capabilities. Gastrointestinal and other infectious diseases, determined by a household and community's access to adequate water, sanitation, hygiene, and health services, increase nutritional needs and can further exacerbate malnutrition.^5^, ^6^ Poor diets drive malnutrition in early childhood.^2^

Even though the stunting prevalence has demonstrated minimal progress in Ethiopia,^7^ it imposed significant devastating impacts on individual health and community development.^8^, ^9^ Despite the execution of national nutrition programs and other programs by the government and bilateral organizations, stunting in Ethiopia is still a serious public health problem.

The global commitment to end all forms of malnutrition is addressed through the vivid application of nutrition intervention, which is comprised of nutrition‐specific and ‐sensitive interventions. Nutrition‐specific interventions refer to interventions that address the immediate determinants of malnutrition while nutrition‐sensitive interventions are designed for health interventions that influence the underlying determinants of nutrition.^10^ Promoting the agricultural sector with a view of nutrition‐sensitive productivity will assist in accelerating nutritional gains in the community, and enhance the production of diverse crops that enrich the quality of diets including micronutrient‐rich foods.^10^ Home gardens promote the production of nutrient‐rich fruits and vegetables that can grow well in local conditions.^11^ For appreciable health and nutrition impact, where nutrition‐specific intervention alone cannot bring desirable change, nutrition‐sensitive agricultural interventions should be coupled with behavior change communication campaigns. These all collectively improve knowledge on the importance of a healthy diet, dietary diversity, and best practices for food preparation, child feeding, and health‐seeking behavior.^12^ In Ethiopia, the available intervention strategies for the reduction of malnutrition are mainly focusing on direct nutrition‐specific programs and poor integration with nutrition‐sensitive agriculture such as supporting the livelihood of rural households, promoting gender equality and nutrition education. The current intervention introduced the provision of egg‐laying pullets, some ingredients for complementary food preparation, and vegetable seeds in addition. This study aimed to evaluate the contribution of nutrition‐sensitive agricultural interventions (home gardening and egg consumption) combined with behavioral‐based nutrition and health education packages on the child feeding practices, hygiene and that leads to reduction of stunting in the rural area of Dessie, Ethiopia through the integration of community extension agents with the community.

## 2 METHODS AND MATERIALS

### 2.1 Intervention development

We established multidisciplinary experts from agriculture, health, communication, and social protection sectors to bring a behavioral change to prevent malnutrition and to have healthy individuals.

Given the causes of malnutrition by its nature are multifaceted and intertwined^13^; we planned to design the intervention that integrates both nutrition‐specific and nutrition‐sensitive agriculture activities. Hence, the intervention process and the Health Belief Model (HBM)^14^ provided the conceptual framework for the design and development of detailed compartments of the intervention packages. The intervention framework integrates theory, practical evidence, and verifiable findings from reports and information collected from the target population to develop culturally appropriate and empirically sound interventions.^15^

The six steps of intervention mapping were properly done to evaluate whether the goals of the intervention were achieved or not. Then we evaluated the intervention performance and objective alignment by contextualizing local area features regarding knowledge, behavior, predisposing, and enabling factors (existing opportunities and threats) that attribute to wide practices of sub‐optimal feeding patterns, deep‐rooted malnutrition, and morbidities. A conceptual framework showing the inter‐linkage between the expected outcomes and mediating factors for the improvement of child growth was modified and developed.^13^, ^16^, ^17^ The best practical evidence from the literature^18^, ^19^, ^20^ and a summary of theoretical methods^21^ were used in the selection of existing methods and techniques. The HBM was used as the focus of behavioral change during the selection of behavior‐based methods and strategies which has been widely employed in health and nutrition behavior change studies.^22^

### 2.2 Study participants and sampling procedure

We implemented the intervention in two selected Kebeles (namely, Abaso Kotu and Kolla Motie) with high malnutrition burden and rural food in‐secured areas of South Wollo Zone, Ethiopia. Lactating mothers with their children below the age of 12 months were recruited to receive the intervention packages and followed up for 12 months. All lactating mothers with infants younger than 12 months and pregnant women and lived for at least 6 months were included in the study. All of the participants who fulfilled the inclusion criteria were registered for the intervention. We carried out adequacy evaluative intervention, which is recommended to compare the outcome in the target group with either previously defined goal or with changes observed in the target group following the intervention program,^23^, ^24^ to measure the contributions of our intervention in improving and sustaining maternal and child nutrition. The baseline survey was carried out in May 2019 and the end line survey was taken in April/May 2020 after 12 months of the period but with the same season.

The intervention was designed, by the end of the intervention program, to achieve the reduction of stunting by 20%. The sample size was determined using STATA version 15 by considering the percentage of change in length‐for‐age *Z*‐score (LAZ) from a study in Malawi.^25^ The baseline mean LAZ score was −1.81 with a SD of 1.1, and the end line SD and correlation were 1.15 and 0.65, respectively. We had also assumed that 95% of confidence level, 80% of power and 10% of loss to follow up. Then the final sample size was found to be 170 mother‐child dyads. During the baseline survey, a minimum of the above sample size was enrolled. However, any woman who delivered after the baseline survey was also registered and provided the intervention packages. As a result, during the end‐of‐intervention survey, there were 270 mother‐child dyads.

### 2.3 Interventions

The interventions were implemented in the first 1000 days plus of critical lifespans. To induce lasting behavior change, ensure continuity, and enhance the linkage between communities and health facilities, secondary target groups consisted of farmers, family members, women development army (WDA), agricultural development army or agent (ADA), health extension workers (HEW), and Kebeles administrators had been incorporated in the intervention.

The nutrition education package was composed of four components, namely (a) education and counseling of mothers or caregivers along with the provision of vegetable seeds and egg‐laying pullets, (b) training of HEWs, ADA, farmers and WDA about diversified food production via organic agriculture, modification, complementary food preparation, and feeding behaviors. (c) Bimonthly home visits and supervision of community‐based nutrition mentors (HEWs and ADA), (d) sensitization workshops, meetings, food festivals, and experience‐sharing through the community‐field visits. Draft versions of the package were discussed with ‐district health and nutrition experts, village health committees and fine‐tuned by the research team.

The intervention primarily applied nutrition and health education intervention that aimed to change feeding behavior, hygiene conditions, improving livelihood status, diversified diet, and nutrition. Frontline agents (HEWs and ADA) compiled all lists of program beneficiaries and called to participate in nutrition education which was voluntary and consent was sought from the caregivers before the commencement of the sessions. The intervention included five sessions (see Table S1) with both group training and demonstration.

The education was designed based on the findings obtained from the baseline survey and reshaped in accordance with recommendations from the Food and Agricultural Organization (FAO),^26^ World Health Organization (WHO),^27^ and United States of Children's Fund (UNICEF)^28^ training resources. We developed the training materials both in the local language (Amharic) and the English language. Key messages on organic production, breastfeeding, complementary feeding, hygiene, and nutritious diet were published and distributed in the form of leaflets, brochures, posters, and manuals and the food pyramid was used to describe local available food groups customized from the national nutrition strategies of Ethiopia^29^ and during each session, every participant was given posters, leaflets, and manuals to be used at their residential address. We made individual home‐based follow‐up sudden visits to assess the adoption of all participants to the training given following each session, to reinforce appropriate practices, and to correct mal‐practices. The follow‐up visits were conducted by the researcher and respective health workers and developmental agents from the intervention villages.

In addition to nutrition education, we also provided at least six egg‐laying pullets, five types of vegetable seeds (cabbage, tomato, carrots, lettuce and spinach), and varieties of complementary foods (legumes, nuts, and some cereals), which are not produced in the areas but have the potential for intensified production in these environs. The success of the nutrition education package was evaluated via baseline and end‐line survey findings. Extensive process evaluation was also being performed to document the reach, dose, and fidelity of the intervention.

### 2.4 Data collection and measurements

The baseline survey questionnaire, with additional questions targeting the nutrition education intervention, was administered during interviews with the caregivers. We recruited six data collectors and trained them on how to gather the information.

The questionnaire comprised of socio‐demographic characteristics, the socioeconomic conditions, food security status, morbidity occasions, child breast and complementary feeding practices, maternal behavior regarding child feeding, water, sanitation and hygiene (WASH), and child anthropometric indices. The socio‐economic status of the households was assessed by computing the wealth index based on the housing condition, the main source of drinking water, types of latrine, the main type of fuel, land ownership, and household assets (electricity, television, radio, watch, phone, bed, car, wagon, bicycle, bank account, and goat/sheep/cow/ox/horse).

We performed principal component analysis to classify the economic status into quintiles.^30^ The change in food security status was also measured by the validated tool of the FAO guideline to categorize as food secured, mildly, moderately, and severely food secure.^31^, ^32^ To measure the difference in the child and maternal hygiene before and after the intervention, we assessed WASH components using nine‐item questions with a score of one for those who fulfilled recommendations and zero for those who did not practice it properly. The WASH index was computed using the principal component analysis to divide the hygienic condition of children and mothers into terciles.^33^ The contributions of the intervention on maternal or caregivers' behavioral change on feeding practice were assessed using the HBM,^34^ which contained six components: perceived susceptibility, perceived severity, perceived benefit, perceived barriers, cue to action, and self‐efficacy. To identify any observed change in behavior in the six constructs, we ran confirmatory factor analysis and judged based on the differences detected from the baseline findings.

The child dietary diversity score (CDDS) and minimum dietary diversity for woman (MDD‐W) were determined from the 24‐hours recalls.^35^, ^36^ The CDDS score ranges from zero to eight, and children who eat foods from five or more food groups daily were met the minimum recommended dietary diversity.^35^ The variety of foods consumed by the children was further analyzed in detail using 14 food items. We also computed age‐specific infant and young child feeding (IYCF) indicators such as early initiation of breastfeeding, exclusive breastfeeding (EBF), initiation of complementary food, minimum meal frequency (MMF), minimum dietary diversity (MDD), minimum acceptable diet (MAD), and the proportion of children who consumed iron‐rich food, continued breastfeeding and malpractices related to child feedings. MMF is defined as the proportion of breastfed and non‐breast‐fed children 6 to 23 months of age who receive solid, semi‐solid, or soft foods (including milk feeds for non‐breast‐fed children) the minimum number of times or more the previous day. The MMF was defined as two times for breastfed children aged 6 to 8 months, three times for breastfed children aged 9 to 23 months, and four or more times for non‐breastfed children aged 6 to 23 months. MAD is a composite indicator calculated from two fractions: breastfed children 6 to 23 months of age who had at least the minimum dietary diversity and minimum meal frequency during the previous day; and non‐breast‐fed children 6 to 23 months of age who received at least two milk feedings and had at least the minimum dietary diversity not including milk feeds and the minimum meal frequency during the previous day.^35^ MDD‐W was determined using a guide recommended by Food and Nutrition Technical Assistance Version‐3 (FANTA‐III) from grains, roots, and tubers; pulses; nuts and seeds; dairy; meat, poultry, and fish; eggs; other Vitamin A‐rich fruits and vegetables; dark leafy greens and vegetables; other vegetables; and other fruits.^36^ The woman consumed five and above food groups per 24‐hours of duration met the minimum recommendation.

To assess child nutritional status, weight, mid‐upper arm circumference (MUAC) and length were measured during the baseline, monthly on the subsequent follow‐up periods, and end‐line survey. Weight was taken using a well‐calibrated and adjusted salter scale in the kilogram to the nearest 10 g and one digit after decimals. Recumbent length in centimeters was measured with a portable wood‐made sliding board and recorded to the nearest 1 mm. MUAC was taken at the mid‐point of the right arm of each child as recommended by WHO. Each measurement was obtained twice and results were averaged. If results were >3% discrepant, then a third measurement was obtained. Results were converted to *Z*‐scores using WHO Anthro.^37^

### 2.5 Data analysis

Data were managed via digitalized electronic devices; stored as a form of comma‐delimited file and exported to STATA/SE version 15 (StataCorp LP, College Station, Texas) for analysis. Frequency distributions were done to identify outliers. The descriptive statistics were done separately for the baseline and end‐line data sets (meaning 170 mother‐child pairs at baseline and 270 mother‐child pairs at end line) (Table 1). However, for other statistical tests (paired *t* test, *χ* ^2^ test, structural equation modeling (SEM) and general linear model (GLM) paired data set was used to detect the change. Changes in socio‐demographic characteristics, food groups consumed, feeding behavioral change between the baseline and end‐line were tested using a Paired *t* test for continuous variables, and *χ* ^2^ test for nominal variables. Individual dietary diversity scores of children and women were calculated according to WHO specifications of 8 and 10 food groups, respectively.^35^, ^36^

**TABLE 1:** Percentage distributions of target intervention participants in rural District of Dessie, North Central Ethiopia 2020

Characteristics	Baseline (n = 170)	End line (n = 270)	*P*‐value
	Frequency (%)	Frequency (%)	
Marital status			
Married	161 (94.7%)	258 (95.5%)	.75
Divorced/widowed	9 (5.3%)	12 (4.5%)	
Place of resident			
Abaso Kotu	100 (58.8%)	160 (59.2%)	.0006
Kolla Motie	70 (41.2%)	110 (40.8%)	
Educational status			
Cannot read and write	50 (29.4%)	80 (29.6%)	.25
Can read and write	38 (22.3%)	60 (22.2%)	
Primary	52 (30.6%)	98 (36.3%)	
Secondary and above	29 (17.7%)	32 (11.9%)	
Maternal age (y)			
Mean ± SD	28.3 ± 5.4	28.5 ± 6	.21
Family size			
Mean ± SD	5.1 ± 1.6	5.4 ± 2.2	.14
Child age (mo)			
0–5	62 (36.4%)	48 (17.7%)	
6–23	108 (63.6%)	222 (82.3%)	
Mean ± SD	6.5 ± 3.4	11.6 ± 5.8	.0007
Child sex			
Male	92 (54.1%)	139 (51.5%)	.13
Female	78 (45.9%)	131 (48.5%)	
Food security			
Food secured	96 (57.3%)	169 (62.5%)	.085
Mildly insecure	45 (26.1%)	61 (22.3%)	
Moderately insecure	20 (11.5%)	34 (12.3%)	
Severely insecure	9 (5.1%)	6 (3.7%)	
ANC follow up			
Yes	159 (93.3%)	255 (94.4%)	.003
No	11 (6.7%)	15 (5.6%)	
Iron‐folic acid supplementation			
Yes	102 (60.0%)	172 (63.4%)	.41
No	68 (40.0%)	98 (36.6%)	
Post natal follow up			
Yes	165 (97%)	256 (94.8%)	.63
No	5 (3%)	14 (5.2%)	
Place of delivery			
Home delivery	32 (18.8%)	27 (10%)	.65
Health facility	138 (81.2%)	243 (90%)	
Child vaccination			
Yes	157 (92.3%)	263 (97.4%)	.15
No	13 (7.7%)	7 (2.6%)	
Vitamin A supplementation			
Yes	114 (67.1%)	225 (83.3%)	.09
No	56 (32.9%)	45 (16.7%)	
Zinc supplementation			
Yes	42 (24.7%)	64 (23.7%)	.098
No	128 (75.3%)	206 (76.3%)	

Reliability analysis was performed for composite variables such as wealth index, WASH index, maternal behavior on child feeding and anthropometric measurements. Cronbach's alpha was used to assure the internal consistency of items which was greater than 0.7 for each composite variable. We also checked coefficient of variations, coefficient of reliability and technical error of measurement (TEM) for evaluating the validity of anthropometric measures.

Anthropometric indices such as weight‐for‐length *Z*‐score (WLZ), length‐for‐age *Z*‐score (LAZ), weight‐for‐age *Z*‐score (WAZ), Body mass index‐for‐age *Z*‐score (BAZ) and mid‐upper arm circumference‐for‐age *Z*‐score (MUACZ) were generated using WHO Anthro version 3.2.2 growth standard.^31^ Biologically implausible values based on WHO‐recommended cutoffs at 6 SD were eliminated. The prevalence of stunting, wasting, and underweight were compared between the baseline and end‐line. Changes in the mean score of the LAZ, WLZ, and WAZ were tested using a paired *t* test. The current average annual rate of reduction of stunting (AARR) was determined as AARR = 1 − (*P*_*t*+*n*_/*P*_*t*_). Where *P*_*t*+*n*_ is the latest prevalence of stunting after 1 year, *P*_*t*_ is the starting year prevalence of stunting, and *n* is the number of years between them.

SEM was used to predict the status of child stature; path analysis (confirmatory factor analysis [CFA]) was utilized to assess the direct and indirect relationships of the observed and unobserved variables of health belief constructs of HBMs with child growth. The analysis was managed using AMOS 23. The health beliefs, feeding behaviors, and hygiene conditions were investigated for the presence of a mediator effect on child malnutrition. The degree of correspondence between the conceptual model and actual data was evaluated using a good‐of‐fit test. The cut‐off criteria to consider the model a good fit to the data included CFI >0.90, TLI >0.90, RMSEA and a standardized root mean square residual (SRMR) <0.06.^38^ There were modest increases in the factor loadings of items that fulfill the assumption of CFA, each of them was greater than 0.7 (GFI = 0.913, CFI = 0.97, TLI = 0.96, RMSEA = 0.048 and SRMR = 0.036). Higher factor scores of observed and unobserved variables were seen with end‐line survey result which has a relatively higher number of participants.

General linear models with random intercepts and robust standard errors were used to assess for continuous repeated LAZ‐score of children between the baseline and end‐line data.

The effects of covariates for the difference in child LAZ (T1‐T2) were evaluated through this model with the repeated measures of analysis of variance (ANOVA) at 95% of confidence level and coefficient of determination. The statistical significance level was declared at a *P*‐value of less than .05.

## 3 RESULTS

### 3.1 Characteristics of study participants

The intervention began with 170 mother‐child pairs, and after a year, the project beneficiaries had grown 270 mother‐child pairs. At baseline and end‐line, the mean age (SD) of mothers was 28.28 (SD = 5.4) and 28.50 (SD = 5.98) years, respectively. The basic demographic, economic features and health service utilization of study participants at baseline and end‐line survey were summarized in Table 2 below. The average ages of children at baseline and end line were 6.5 (SD = 3.4) and 11.6 (SD = 5.8) months. The research participants' food insecurity levels differed from the baseline data, which showed a 5.2% reduction in food insecurity. Some incremental changes in health service coverage were observed in terms of antenatal care follow‐up, Iron‐Folic acid supplementations, child vaccinations, vitamin A and Zinc supplementations (Table 1).

**TABLE 2:** The mean scores of items at baseline and endline survey and paired differences of mean scores after intervention for items that retained in the model to measure changes in feeding behaviours of mothers in North Central Ethiopia 2020 (n = 170)

Items of subscale	Mean (SD)		Paired difference	*P*‐value (two‐sided)	Item‐total correlation	Cronbach's *α* If item deleted (n = 170)	Cronbach's *α* of the subscale (n = 170)
	Baseline	Endline					
Subscale of perceived susceptibility							.80
Do you perceive that eating monotonous diet lead to malnutrition?	3.81 (0.81)	4.01 (0.70)	0.202	**.006**	0.61	.74	
If appropriate balanced diet is not consumed during pregnancy, do you perceive that fetus will get malnourished?	3.94 (0.59)	4.06 (0.70)	0.116	.056	0.53	.78	
You feel that you and your fetus are at risk of developing complications as a result of inadequate intake of balanced diet?	3.97 (0.55)	4.09 (0.65)	0.116	**.034**	0.65	.72	
Do you perceive that taking alcohol during pregnancy lead to malnutrition?	3.73 (0.81)	4.05 (0.71)	0.314	**.00001**	0.64	.73	
Subscale of perceived severity							.88
Do you perceive that maternal malnutrition during pregnancy will cause still birth?	4.01 (0.57)	4.05 (0.75)	0.043	.485	0.70	.86	
Do you perceive that if mothers malnourished during pregnancy, their child likely to die?	4.05 (0.59)	4.08 (0.72)	0.035	.556	0.64	.87	
Do you feel that you will get malnourished sometime during your pregnancy stage?	3.97 (0.65)	4.11 (0.72)	0.140	**.033**	0.65	.87	
Do you afraid to think about malnutrition during pregnancy?	3.95 (0.74)	4.06 (0.78)	0.112	.112	0.69	.86	
Do you perceive that malnourished women during pregnancy are likely to die during delivery?	4.00 (0.60)	4.10 (0.74)	0.101	.112	0.66	.87	
Do you feel that if you are malnourished scares you and your family?	3.92 (0.66)	4.03 (0.71)	0.112	.080	0.65	.87	
Do you perceive that if mothers malnourished during pregnancy, their child born with low birth weight?	4.01 (0.69)	4.14 (0.53)	0.132	**.027**	0.69	.86	
Subscale of perceived benefits							.82
Do you think that preparing hygienic foods prevents you from infection?	4.05 (0.46)	4.2 (0.63)	0.151	**.004**	0.65	.77	
Do you think that when you prepare meals from variety of foods, you are doing something to take care of yourself as well as your child?	3.96 (0.50)	4.05 (0.63)	0.089	.096	0.71	.75	
Do you think that preparing food from varieties of food sources such as animal and plant food sources decrease the chance of developing malnutrition?	4.02 (0.51)	4.11 (0.66)	0.085	.135	0.65	.78	
Do you think that appropriate dietary habits during pregnancy will decrease the likely of low birth weight and death of your child?	4.05 (0.49)	4.09 (0.74)	.035	.553	0.58	.81	
Subscale of perceived barriers							.87
Do you perceive that preparing meal from variety of food laborious?	2.71 (1.14)	2.80 (1.18)	0.089	.341	0.79	.81	
Do you perceive that eating extra meals during pregnancy will challenge during delivery?	3.08 (1.06)	2.95 (1.17)	−0.136	.174	0.68	.85	
Do you perceive that preparing balance diet is too costly?	3.29 (1.06)	3.10 (1.17)	−0.190	**.039**	0.73	.83	
Do you perceive that preparing meal from variety of food time consuming?	2.74 (1.11)	2.89 (1.17)	0.155	.125	0.63	.86	
Do you perceive that eating extra meals during pregnancy will influence pregnancy outcome?	3.27 (1.00)	3.19 (1.15)	−0.074	.447	0.65	.85	

Items of subscale	Mean (SD)		Paired difference	*P*‐value (two‐sided)	Item‐total correlation	Cronbach's *α* If item deleted (n = 170)	Cronbach's *α* of the subscale (n = 170)
	Baseline	Endline					
Subscale of cues to action							.97
Transmission of malnutrition to the new born motivates you to take care of my nutrition.	4.03 (0.50)	3.86 (1.14)	−0.167	.056	0.86	.97	
Fears of low birth weight due to malnutrition motivate you to take diversified diet regularly.	4.00 (0.44)	3.77 (1.16)	−0.225	**.008**	0.91	.97	
Chronic consequence of malnutrition to you and my new born motivate you to prevent malnutrition.	4.01 (0.44)	3.84 (1.18)	−0.174	**.041**	0.92	.97	
Fear of malnutrition as a complication motivates you to adhere on eating diversified diet regularly.	3.97 (0.58)	3.81 (1.18)	−0.159	.077	0.93	.96	
Your family's and friends' advice motivates you to take diversified diet regularly.	4.03 (0.52)	3.79 (1.20)	−0.236	**.009**	0.95	.96	
Health and nutrition education about pregnancy specific nutrition motivates you to eat diversified diet and to prevent malnutrition.	3.64 (0.99)	3.80 (1.09)	0.155	.114	0.92	.97	
Subscale of self‐efficacy							.96
I can eat meal with appropriate diversity.	3.95 (0.43)	3.71 (1.19)	−0.248	**.003**	0.89	.95	
I can eat meal with appropriate amount.	3.97 (0.44)	3.65 (1.24)	−0.322	**.0001**	0.94	.93	
I can eat meals frequently including snakes.	3.95 (0.51)	3.72 (1.20)	−0.233	**.007**	0.89	.95	
I can prepare meals hygienically?	4.10 (0.39)	3.93 (1.18)	−0.163	.052	0.89	.95	

*Note*: Bold values indicates the level of statistical significance at *p*‐value of < 0.05.

### 3.2 Maternal feeding behavior

Constructs of the HBM were used to assess the maternal feeding behaviors and awareness level on the impact of malnutrition. Six constructs, namely, perceived susceptibility, perceived severity, perceived benefit, perceived barrier, cue to action, and self‐efficacy were used to measure the effect of nutrition education on changes in feeding behaviors of mothers.

A 38‐item with Likert scale was analyzed using CFA to examine the direct and indirect effect of the constructs on the child's nutritional status. There was a change in total variance explained between baseline and end‐line characteristics (31.58% vs 38.9%) (see Table S2).

The change responses of item scores for each component of the HBM were tested using paired‐test statistics of the baseline and end‐line findings. The mean difference in item scores for the majority of the items under perceived susceptibility, severity, and benefit showed positive deviance from the baseline, which was significant at *P* < .05, whereas the change in the score of items under cue to action and self‐efficacy, were negative and had an association at *P* < .05.

This indicates the change of maternal behaviors in terms of perceived susceptibility, severity, and benefit had positive improvement in the mean of the item scores, but item scores at the end line were decreased in the responses of the cue to action and self‐efficacy (Table 2).

### 3.3 Child feeding characteristics

**FIGURE 1:**
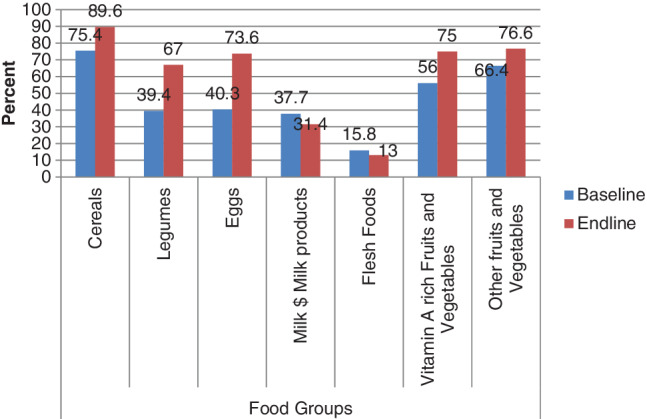
Comparision of percentage of consumption of food groups by children below the age of 24 months in North‐Central Ethiopia 2020

There were no significant changes between baseline and end line survey in the percentages of food groups such as cereals (75.4% vs 89.6%, *P* = .82), milk and milk products (37.7% vs 31.4%, *P* = .19), fresh food (15.8% vs 13%, *P* = .38), and vitamin A rich fruits and vegetables (56% vs 75%, *P* = .06), as shown in Figure 1. However, after the provision of some selected complementary food ingredients, egg‐laying pullets and vegetable seeds, significant improvement in the percentage of food groups consumption were observed in legumes (39.4% vs 67%, *P* = .021), eggs (40.3% vs 73.6%, *P* = .001) and other fruits and vegetables (66.4% vs 76.6%, *P* = .04).

**FIGURE 2:**
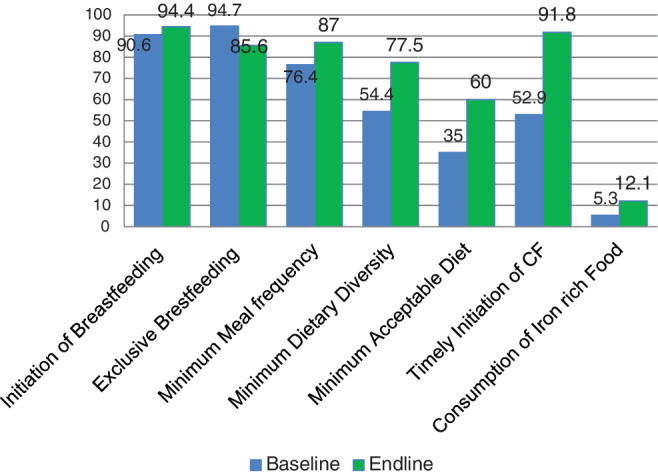
Percentage of WHO‐IYCF practices of children under the age of 2 years before and after the intervention in North‐Central Ethiopia 2020

At the start of the study, 54.4% of youngsters fulfilled the minimal dietary diversity recommendation, and at the end, 77.5% did. Out of pair of 111 children, the mean dietary diversity score was significantly higher at the end‐line (5.12) than the baseline (4.03), a difference of 1.1 and a *P*‐value <.003 (Table 3). We compared the WHO‐IYCF indicators as illustrated in Figure 2 and no difference was detected in the initiation of breastfeeding within 1 hour of giving birth (90.6% vs 94.4%, *P* = .23) and the percentage of exclusive breastfeeding (94.7% vs 85.6%, *P* = .06). Nevertheless, among the IYCF core indicators, minimum dietary diversity (54.4% vs 77.5%, *P* = .013), MAD (35% vs 60%, *P* = .002) and initiation of complementary food (52.9% vs 91.8%, *P* = .004) showed significant improvement after the intervention had been given.

**TABLE 3:** The mean differences of children's and mothers' feeding practices and nutritional status in North‐Central Ethiopia 2020 (n = 170)

Characteristics	Baseline	Endline	Mean difference (95% CI)	*t*	*P* ^£^‐value
	Mean (±SD)	Mean (±SD)			
Child characteristics					
Weight (kgs)	7.18 (1.49)	8.06 (1.97)	−0.89 (−1.37, −0.41)	−3.7	.0003
Height (cm)	63.94 (11.75)	70.82 (10.18)	−6.87 (−9.90, −3.85)	−4.5	.00018
WLZ	0.43 (3.34)	−0.63 (1.86)	1.06 (0.40, 1.70)	3.2	.002
LAZ	−1.18 (3.46)	−0.45 (1.78)	−0.73 (−1.40, −0.06)	−2.1	.034
WAZ	−0.78 (1.21)	−0.79 (1.41)	0.01 (−0.31, 0.33)	0.1	.93
BAZ	0.10 (2.82)	−0.73 (1.82)	0.84 (0.26, 1.42)	2.9	.005
MUAC (cm)	12.11 (0.65)	13.03 (1.91)	−0.93 (−0.91, −0.36)	−3.5	.001
MUACZ	−2.01 (0.77)	−1.38 (0.85)	−0.63 (−1.46, −0.40)	−4.6	.00001
CDDS	4.03 (3.21)	5.12 (1.87)	−1.1 (−2.09, −0.10)	−2.2	.003
Mothers characteristics					
Weight (kgs)	52.0 (6.94)	55.4 (8.50)	−2.6 (−0.25, 4.63)		.08
MUAC (cm)	21.32 (1.78)	22.09 (2.82)	−0.77 (−1.22, −0.32)	−3.4	.001
MDD‐W	5.35 (1.95)	5.85 (2.28)	−0.49 (−0.86, −0.13)	−2.7	.008

*Note*: The symbol ‘£’ represents a *p*‐value < 0.05.

### 3.4 Child nutritional status

**FIGURE 3:**
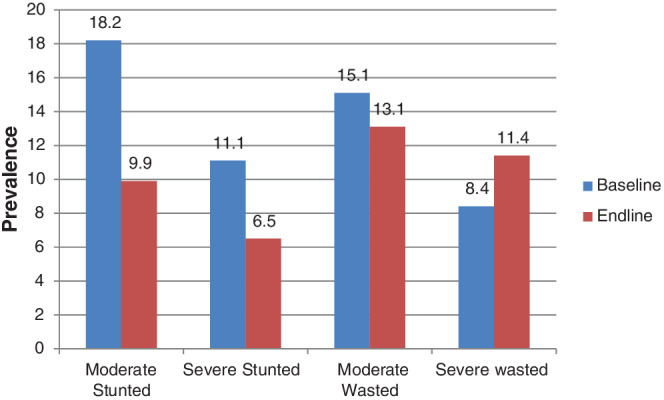
The degree of acute and chronic child malnutrition before and after the intervention in North‐Central Ethiopia May 2019 to May 2020

From a total of 170 under 2 years of children, the prevalence of acute malnutrition (wasting, WLZ <−2 SD) and stunting (LAZ <−2 SD) at the baseline were 23.5% (95% CI = 11.1, 32.8) and 29.3% (95% CI = 18.6, 42.7), respectively. One year later, among 270 children the prevalence of wasted children almost remained the same (24.5%) (95% CI = 17.6, 33.7) but the prevalence of chronically malnourished children was found to be 16.4% (95% CI = 10.7, 24.2) (Figure 3).

Girls and older children (age greater than 5 months) were more affected by stunting at both baselines and end‐points. When we compared changes in child anthropometric profiles of 170 children, who have a baseline and end‐line data, there were improvements in children's weight, height, MUAC, LAZ, and MUAC‐for‐age (all significant at *P* < .05) (Table 3).

### 3.5 Predictors of child linear growth

The contribution of intervention had shown a substantial increment in the average LAZ score of children (paired‐sample *t* test = −2.1, *P* = .034). The project employed nutrition‐sensitive interventions such as nutrition education to change feeding behaviors of children and mothers and egg production for sustainable consumption. The independent effects of each of the components of interventions on childhood linear growth were determined by using the general linear model for the repeated measures of ANOVA (Table 4). Among all covariates adjusted for the model, only consumption of eggs on a daily basis influenced the LAZ score of children.

**TABLE 4:** General linear model showed the effect of nutrition‐sensitive intervention on child linear growth in North‐Central Ethiopia May 2020 (n = 170)

Outcome variable	Explanatory variables	*B* (95% CI)	*P*‐value	Partial eta squared
L/HAZ score at Baseline	Intercept	2.19 (−11.02, 8.13)	.52	0.012
	Factor score of perceived susceptibility	0.29 (−1.72, 2.31)	.74	0.012
	Factor score of perceived severity	−0.89 (−3.65, 1.86)	.48	0.056
	Factor score of perceived benefit	−0.68 (−3.67, 2.32)	.62	0.028
	Factor score of perceived barrier	1.13 (−0.98, 3.23)	.26	0.139
	Factor score of cue to action	0.89 (−1.42, 3.20)	.41	0.077
	Factor score of self‐efficacy	−0.31 (−2.43, 1.81)	.75	0.012
	Consumption of eggs	0.43 (−7.92, 8.78)	.91	0.002
	Child dietary diversity score	−0.20 (−1.46,1.06)	.73	0.014
	Age of child	0.05 (−0.72, 0.82)	.88	0.003
	Minimum dietary diversity for Women	0.25 (−1.52, 2.02)	.75	0.011
L/HAZ score at endline	Intercept	−2.98 (−11.20, 5.24)	.43	0.070
	Factor score of perceived susceptibility	−0.03 (−0.81, 0.77)	.94	0.001
	Factor score of perceived severity	0.01 (−1.76, 1.76)	.99	0.000
	Factor score of perceived benefit	1.03 (−0.24, 2.32)	.10	0.270
	Factor score of perceived barrier	−0.60 (−1.64, 0.43)	.23	0.163
	Factor score of cue to action	−0.60 (−4.63, 3.41)	.74	0.012
	Factor score of self‐efficacy	−0.18 (−3.50, 3.16)	.90	0.002
	Consumption of eggs	**1.12 (1.04, 5.23)**	**.042**	**0.364**
	Child dietary diversity score	0.87 (−0.39, 2.12)	.15	0.212
	Age of child	−0.06 (−0.44, 0.30)	.69	0.018
	Minimum dietary diversity for Women	0.31 (−0.36, 0.97)	.32	0.109

*Note*: Bold values indicates the level of statistical significance at *p*‐value of < 0.05. The model was adjusted for child sex, maternal age and education, economic status and WASH.

Consumption of an egg per day increases the child's LAZ score by1.12 units after 1 year of intervention. The variability of LAZ score after 1 year was, explained by the partial error term squares; egg consumption adjusted for other covariates changed by 36.4% (Table 4). However, none of the HBM constructs showed a significant effect on child growth.

## 4 DISCUSSION

The research was aimed at reducing the burden of childhood stunting by combining nutrition and health education with a variety of other nutrition‐specific and nutrition‐sensitive interventions. The prevalence of stunting declined in the project area from 29.3% (95% CI = 18.6, 42.7) at baseline in May 2019 to 16.4% (95% CI = 10.7, 24.2) at the end‐line in May 2020. The average annual reduction rate of stunting was 44%, indicating that the intervention was successful in meeting the study target. As a general rule, programs with an average annual reduction rate (AARR) of stunting of at least 3 (median AARR) from the baseline were considered effective.^39^ This change was comparable with findings from a systematic review of interventional studies which ranged from 4.3% to 8.4%.

In this project, nutrition‐sensitive agriculture in combination with nutrition‐counseling and specific interventions has shown a positive impact on child stature. Increasing agricultural production for human consumption helps to reduce stunting in children under the age of 2 years. The rate of thawing was best explained by eating at least one egg per day and having a higher kid dietary diversity score. Studies show that diets involving eggs significantly improve child growth,^40^ because the egg is an excellent source of these nutrients (essential amino acids, lipids, and choline which play a significant role in child growth and development).^41^ A randomized controlled trial in Ecuador revealed that the early introduction of eggs into child complementary foods reduced stunting by 47% (43) which supports the current findings that egg consumption increased LAZ‐score by almost one unit (responsible for 36% of LAZ score increment). Furthermore, a recent review concludes that egg consumption affects not only the child's linear growth but also has a significant impact on brain development.^43^ However, an experimental study in Bangladesh found that providing a daily egg to infants for 6 months had an effect on ponderal but not linear growth^44^ and trials in Malawi and Zambia found that providing 1 egg per day to children had no overall effect on linear growth.^45^ This finding was also congruent with the evidence that consumption of generic animal source foods (ASF) (dairy, meat/fish, and egg) has a strong statistical association with stunting and such that consuming multiple ASFs is more advantageous than any single ASF.^46^ These variations may be due to variability in the intervention period (short follow‐up time), study period difference, relatively dose of egg consumption, and some other methodological limitations as partly reflected in the study conducted in Ecuador and Zambia suggested the need for a longer intervention period and ongoing nutrition support to young children during early childhood and egg consumption has no significant effect on the child linear growth after the age of 2 years.^42^, ^47^, ^48^

Despite the lack of clear evidence on the impacts of agriculture on nutritional status, most vegetable gardening treatments have shown a beneficial effect on dietary intake and nutritional status measurements such as anthropometric indicators when combined with behavioral modification interventions.^49^ Agriculture improvements that are supported by nutrition education are likely to be successful.^50^ A review of high impact literature of agricultural interventions and strategies from agricultural organizations depicted those largely home gardens with animal production, reveals a positive impact on consumption of vitamin A‐rich fruits and vegetables, while the evidence of the positive impact of targeted agriculture interventions on maternal and child nutrition is limited.^51^ Nutrition‐sensitive agricultural projects (in particular home gardening, egg production) in Nepal and Bangladesh demonstrated significantly higher consumption of special foods such as eggs, meat, milk, nuts, and dried fruits, but no data on the impact of such foods on child anthropometric status was found.^11^, ^51^

Analysis of the situation in South Asia suggests that in the first 1000 days, it is critical to combine nutrition‐sensitive therapies with a package of evidence‐based direct nutrition interventions.^26^, ^52^ Women's empowerment and gender equality have a significant impact on women's health and nutrition. According to a recent report, a reduction in gender inequality would be beneficial, which could contribute to a 10 percentage points decline in stunting prevalence rate in children.^53^

The study applied path analysis to identify direct and indirect associations between feeding behaviors and child linear growth. It also implemented intervention strategies that were devised taking into account community‐specific concerns discovered through a variety of ways. The study's limitations include the inability to determine if the improvement was an independent consequence of the intervention we applied. It did not take into account the intervention group's changes in contrast to the other control groups and the existence of governments and non‐government intervention programs in the area may mask the realistic intervention effect on child growth. Quantitative estimation of child nutrient intakes and micronutrient deficiencies were not assessed in the study.

## 5 CONCLUSIONS

We concluded that the combination of nutrition‐sensitive agriculture and nutrition‐specific interventions improve child growth. A child's linear growth outcome by LAZ was reduced by about 13% from the baseline finding. Adjusted by sex, age, and some other variables, the daily basis of egg consumption during the first 1000 days significantly improved linear growth and reduced stunting. Socio‐cultural phenomena, maternal and child feeding behavior, individual diversity, access to water, sanitation and hygiene, food insecurity, morbidity status, and health service utilization did not significantly impact child stature but may have a mediator effect on the pathway of malnutrition.

Given the complex nature of these diverse intervention packages, strong political commitment, multi‐sectoral collaboration, community‐based service delivery platforms, and wider program coverage and compliance are all likely critical components of effective stunting reduction programs.

For the rapid reduction of stunting prevalence in children in developing countries, strengthening nutrition‐sensitive agriculture, in particular, access and availability of agricultural production (eg, gs, vegetables, fruits, and nuts), behavioral change interventions, improving the socio‐economic situation and decision‐making power of women must complement with the ongoing efforts of improving coverage of the direct nutrition interventions. Moving forward, the study calls for taking a strong commitment to scaling the intervention to the larger rural community; nutrition‐sensitive intervention, especially sustainable egg consumption (which have the potential to be an affordable and environmentally sustainable high‐quality food source in populations at risk for malnutrition) during the first 2 years of life. The provision of multiple intervention packages at the same time promotes stunting reduction in order to meet the target of the Sustainable Development Goals of ending all forms of malnutrition by 2030.

## FUNDING

Wollo University and Institute for Sustainable Development (ISD) were funded the research. The funders had no role in study design, data collection and analysis, decision to publish, or preparation of the manuscript.

## CONFLICT OF INTEREST

The authors declare that they have no competing interests related to authorship and financial issues.

## AUTHOR CONTRIBUTIONS

TM: Conceptualization, Data curation, Formal analysis, Funding acquisition, Investigation, Methodology, Project administration, Resources, Software, Supervision, Validation, Visualization, Writing and review manuscript (Lead); ST: Conceptualization, Data curation, Funding acquisition, Investigation, Methodology, Project administration, Resources, Software, Supervision, Validation, and review manuscript(equal); YD: Conceptualization, Data curation, Formal analysis, Funding acquisition, Investigation, Methodology, Project administration, Resources, Supervision, Validation, Visualization, and reviewing manuscript (equal); NC: Conceptualization, Data curation, Formal analysis, Funding acquisition, Investigation, Methodology, Project administration, Resources, Software, Supervision, Validation, Visualization, Writing and reviewing manuscript (equal); HA: Conceptualization, Data curation, Formal analysis, Funding acquisition, Investigation, Methodology, Project administration, Resources, validation and reviewing manuscript (equal); GS: Conceptualization, Data curation, Formal analysis, Funding acquisition, Investigation, Methodology, Project administration, Resources, Supervision, and reviewing manuscript (equal); FM: Funding acquisition, Investigation, Methodology, Project administration, Resources, Supervision, Visualization, and review manuscript (equal); AH: Funding acquisition, Investigation, Methodology, Project administration, Resources, Supervision, Visualization, and review manuscript (equal).

All authors contributed to data analysis, drafting or revising the article, have agreed on the journal to which the article will be submitted, gave final approval of the version to be published, and agree to be accountable for all aspects of the work.

## TRANSPARENCY STATEMENT

We declare that the scientific report is our original work with appropriate acknowledgements and recognition of others' work used for the development of the manuscript. Parts or whole section of figures, texts, or tables included in this manuscript are not submitted to other journals for publication.

## ETHICS STATEMENT

Ethical clearance was obtained from the ethical review board of Wollo University, College of Medicine and Health Sciences. Permission was obtained from local district leaders. Each study participant was communicated clearly about the objective of the study in order to obtain their verbal consent, their full right to withdraw or refuse to participate. Privacy and confidentiality of information taken from each study participant were handled in a secured manner.

## Supporting information


**Table S1.** Nutrition and health education sessions contents for mothers about behavioral change in feeding practices, hygiene and health conditions.
**Table S2.** Rotated Component factor scores of Health Belief Model items for Malnutrition during pregnancy and first two years of child age of the Baseline and endline survey results in South Wollo, Ethiopia 2020.

## Data Availability

The datasets generated and/or analyzed during the current study are not publicly available due to the lack of databases in our institution but are available from the corresponding author on reasonable request.
