# Supplementary material for: The role of nutrition‐sensitive agriculture combined with behavioral interventions in childhood growth in Ethiopia: An adequacy evaluation study
Source: Health Sci Rep. 2022 Mar 3;5(2):e524. doi: 10.1002/hsr2.524 (PMC8893299; doi:10.1002/hsr2.524)
Supplement: Supplementary file 1 — Table S1. Nutrition and health education sessions contents for mothers about behavioral change in feeding practices, hygiene and health conditions. Table S2. Rotated Component factor scores of Health Belief Model items for Malnutrition during pregnancy and first two years of child age of the Baseline and endline survey results in South Wollo, Ethiopia 2020. [file HSR2-5-e524-s001.docx]

**Additional File one: - Nutrition and health education sessions contents for mothers about behavioral change in feeding practices, hygiene and health conditions.**

| **Sessions** | **Topics** | **Resources** |
| --- | --- | --- |
| **Session one: June to August 2019** | Organic vegetables production(composite development, home gardening, pesticide, postharvest loss, market linkage) | Distribution of egg-lying pullets, Seven types vegetable seeds, compost materials, organic pesticides, leaflets, manuals , counseling cards, Soap, ash, water in a jar or basin, cups with covers |
|  | Eggs production (feeding, disease prevention and management) |  |
|  | Exclusive and continuation of Breastfeeding |  |
|  | Child and maternal personal hygiene(hand washing, toilet utilization, diarrheal diseases and intestinal parasitosis) |  |
|  | Food safety(hygienic preparation, processing, storage and feeding, environmental contamination of toxins) |  |
|  | Demonstrations |  |
| **Session two: August to September 2019** | Importance of Nutrition and Feeding Behaviors of mothers and children( Malnutrition, impact, health diet, critical periods of human life stage, Health Belief model) | Reading manuals, role play |
| **Session Three: September 2019** | Age appropriate complementary feeding(6-23 months)(initiation, Frequency, diversity, responsive feeding) | Cooking utensils, firewood, Ingredients for recipes, Recipe book, Empty seasonal food availability calendar and set of food cards |
|  | Preparation of complementary foods from seven food groups( from locally available and distributed food stuffs), Family meals and how they affect child nutrition, Seasonal food availability calendar |  |
|  | Participatory cooking demonstration |  |
| **Session four: October 2019** | Plant based-foods(Vegetables, fruits, Cereals, legumes and Nuts) | Food samples, utensils and Recipe book |
|  | Animal-source foods(eggs, poultry, meats, fish, milk and other products) |  |
|  | Participatory cooking session four |  |
| **Session five: end of October 2019** | Overall review of all the five sessions and preparation for the graduation session | Guidelines and leaflets |
| **January, 2020-April, 2020** | Monitoring and follow up | Supervision, review meeting and feedback |

**Additional File two: Rotated Component factor scores of Health Belief Model items for Malnutrition during pregnancy and firs two years of child age of the Baseline and endline survey results in South Wollo, Ethiopia 2020.**

| Factor 1(Baseline) | | Factor 2 | | Factor 3 | | | Factor 4 | | | | Factor 5 | | | | Factor 6 | | | |
| --- | --- | --- | --- | --- | --- | --- | --- | --- | --- | --- | --- | --- | --- | --- | --- | --- | --- | --- |
| Cue to action | | Self-Efficacy | | Perceived benefit | | | Perceived Severity | | | | Perceived Barrier | | | | Perceived Susceptibility | | | |
| Item | Score | Item | Score | Item | | Score | Item | | Score | | Item | | Score | | Item | | | Score |
| Cuemotivb | .818 | Efficamoub | .890 | Benhygb | | .772 | Sevliveryb | | .747 | | Barrconsumb | | .945 | | Susbalanceb | | | .839 |
| Cuedietb | .836 | Efficdiversb | .890 | Bensourcb | | .794 | SevLBWb | | .848 | | Barrlaborb | | .945 | | Susdietb | | | .892 |
| Cuediversb | .861 |  |  | Bendietarb | | .809 | SevSBb | | .889 | |  | |  | | Susskippb | | | .858 |
| Cuebornb | .861 |  |  | Benfeedb | | .812 | Sevdieb | | .889 | |  | |  | | Susmalnb | | | .853 |
|  |  |  |  |  | |  | Sevstagb | | .861 | |  | |  | |  | | |  |
|  |  |  |  |  | |  | Sevafraib | | .746 | |  | |  | |  | | |  |
|  |  |  |  |  | |  | Sevfamilb | | .766 | |  | |  | |  | | |  |
| Eigenvalue |  |  |  |  | |  |  | |  | |  | |  | |  | | |  |
| 7.26 |  | 2.86 |  | 2.18 | |  | 1.84 | |  | | 1.44 | |  | | 1.09 | | |  |
| Variance explained |  |  |  |  | |  |  | |  | |  | |  | |  | | |  |
| 31.58 |  | 12.45 |  | 9.46 | |  | 8.00 | |  | | 6.28 | |  | | 4.75 | | |  |
| Factor 1 (Endline) | | Factor 2 | | | Factor 3 | | | Factor 4 | | | | Factor 5 | | | | Factor 6 | | |
| Cue to action | | Self-Efficacy | | | Perceived benefit | | | Perceived Severity | | | | Perceived Barrier | | | | Perceived Susceptibility | | |
| Item | Score | Item | Score | | Item | Score | | Item | | Score | | Item | | Score | | Item | Score | |
| Cuenewborn1 | .965 | Efficiadiversity1 | .966 | | Benhyg1 | .856 | | SevSB1 | | .792 | | Barrlabor1 | | .883 | | Suscepdiet1 | .808 | |
| CueLBWdiet1 | .954 | Efficiaamou1 | .942 | | Benfood1 | .816 | | Severdie1 | | .786 | | Barrdelive1 | | .837 | | Susfetmal1 | .769 | |
| Cueprevmaln1 | .947 | Effeciafreq1 | .940 | | Bensourc1 | .815 | | Sevstage1 | | .781 | | Barrcostly1 | | .797 | | Suscompl1 | .750 | |
| Cuediversif1 | .946 | Effeciahyg1 | .939 | | Bendiet1 | .757 | | Sevdelivery1 | | .759 | | Barrconsum1 | | .782 | | Susalcohol1 | .728 | |
| Cuetivate1 | .942 |  |  | |  |  | | Sevafrai1 | | .753 | | Barroutcom1 | | .763 | |  |  | |
| Cuediet1 | .904 |  |  | |  |  | | Sevfamily1 | | .750 | |  | |  | |  |  | |
|  |  |  |  | |  |  | | SeverLBW1 | | .744 | |  | |  | |  |  | |
| Eigenvalue |  |  |  | |  |  | |  | |  | |  | |  | |  |  | |
| 11.68 |  | 3.89 |  | | 3.25 |  | | 3.03 | |  | | 1.57 | |  | | 1.24 |  | |
| Variance explained |  |  |  | |  |  | |  | |  | |  | |  | |  |  | |
| 38.9 |  | 12.98 |  | | 10.84 |  | | 6.52 | |  | | 5.23 | |  | | 1.57 |  | |

Keynotes:

Benhygb: Do you think that preparing hygienic foods prevents you from infection?

Bensourcb: Do you think that preparing food from varieties of food sources such as animal and plant food sources decrease the chance of developing malnutrition?

Bendietarb: Do you think that appropriate dietary habits during pregnancy will decrease the likely of low birth weight and death of your child?

Benfeedb: Do you think that when you prepare meals from variety of foods, you are doing something to take care of yourself as well as your child?

Barrconsumb: Do you perceive that preparing meal from variety of food time consuming?

Barrlaborb: Do you perceive that preparing meal from variety of food laborious?

Cuemotivb: Transmission of malnutrition to the new born motivates you to take care of my nutrition.

Cuedietb: Health and nutrition education about pregnancy specific nutrition motivates you to eat diversified diet and to prevent malnutrition.

Cuediversb: Fear of malnutrition as a complication motivates you to adhere on eating diversified diet regularly.

Cuebornb: Chronic consequence of malnutrition to you and my new born motivate you to prevent malnutrition.

Efficamoub: I Can eat meal with appropriate amount.

Efficdiversb: I Can eat meal with appropriate diversity.

Sevliveryb: Do you perceive that malnourished women during pregnancy are likely to die during delivery?

SevLBWb: Do you perceive that if mothers malnourished during pregnancy, their child born with low birth weight?

SevSBb: Do you perceive that maternal malnutrition during pregnancy will cause still birth?

Sevdieb: Do you perceive that if mothers malnourished during pregnancy, their child likely to die?

Sevstagb: Do you feel that you will get malnourished sometime during your pregnancy stage?

Sevafraib: Do you afraid to think about malnutrition during pregnancy?

Sevfamilb: Do you feel that if you are malnourished scares you and your family?

Susbalanceb: If appropriate balanced diet is not consumed during pregnancy, do you perceive that fetus will get malnourished?

Susdietb: Do you perceive that eating monotonous diet (single types of food) lead to malnutrition?

Susmalnb: You feel that you and your fetus are at risk of developing complications as a result of inadequate intake of balanced diet?

Susalcohol1: Do you perceive that taking alcohol during pregnancy lead to malnutrition?
